# Supplementary material for: Sequencing Red Fox Y Chromosome Fragments to Develop Phylogenetically Informative SNP Markers and Glimpse Male-Specific Trans-Pacific Phylogeography
Source: Genes (Basel). 2021 Jan 14;12(1):97. doi: 10.3390/genes12010097 (PMC7828831; doi:10.3390/genes12010097)
Supplement: Supplementary file 1 [file genes-12-00097-s001.zip › SacksetalSIrev/SupplementaryInformation1.docx]

**Sequencing red fox Y chromosome fragments to develop phylogenetically informative SNP markers and glimpse male-specific trans-Pacific phylogeography**

Sacks BN, Lounsberry ZL, Rando HM, Kluepfel K, Fain S, Brown SK, and Kukekova AV.

**Supplementary Information 1. Fasta sequences (and Genbank Nos or references) used to synthesize 2x overlapping 80 bp RNA baits for the Canine Y chromosome.**

**>Fragment 03 (**Natanaelsson et al. 2006; Ding *et al.* 2012)

TTGTCTAAAGTTAAAGTCACAGTACATTAACAAACAAATGATCCTCAGTCACAAAATTATAAATGCAGTTTGGGGTGAGAATAAGCAGGAGGGGAGTTAATCCAAACTACAGCAATTAAGTCTTCAAACCAACTTGTGAATAAGATTGTTGATTGTTTCTTTCTTCTTGTGACTTTGAAGCTCCCTTGAATTTTCAGGAGAATCACCAGCTGGTAAAATGTACTGCAAGGTCTTGAAGCTATAAAATACAGTCTGGCTAAGTTACATGTGGTTTCCATATTAACTTAAGGGTGGCTTTGTCGCACAGCAGATTCAGTTGCCCCACCAGTCAACCCCCTGGGAGTTGTAATTTCCTCCATATCCATCACTACTGTAGAAGCCTCCATAACCACCTATCAAAAAATGTTTGAAAACAGAAATTAGTTTAGCCATTTGCCTCTTCTTTCTCTGATAAAAACCTAATTAAAAAAATTCATAGTACTGGATTTTGTATTCTTTGGAAATAAAATTTCTCCGATGAATTATGGTTTAAGAAAAAAGTAAAAAGCACTAAAATACAAAAGGTATTACCTCCACCAAATCCTCTGCTGCTGCTGTGACCACCTCCACCACTACGGCTGTTGCTTGCACGACTACTGCTAAAGCTAGAACTGCTTGAGCCACTACTTTGTCGA

**>Fragment 11 (**Natanaelsson et al. 2006; Ding et al. 2012)

TTATGTTACTTATTGATAGTAATAACTGGCATTTTAACAGCCCTGTTCAATATGATTTTAGATGATGAGCTTTTATCACTAAAATCAAATAATAGAGGATACTTGCTTAGGAAATTGAAAGTCCAGACTAACCCTTGAAATAGTATGACGCTGAGATAAACTGATTAAAACCCTTGGGTGTTTTAATATTGTATAACCTCATCAAAATATGTTATGATAAGATCCACAAGATTCTTCTAGGGTAGGTGTTATACTTCAGATAAATATATATCTTTTCAGTATTTGAAAACAGGTGTTAATAGTTATTATTAGTTGTTCAGCTGGAGCTTTACAAATCATTGTGTTTTCTGTAGTAAGCAATATCCTTATTGTCATCTGGCATAATAATTCTCATGAATATACTGTTCATACTTACTTTCCTTAATATTGAAGGCTTAAAACTGACCACAATTACTCATTAGTTTCCAGCTCTTAAAAATAACTAATTATTGGGACGCCTGTATGACTCCATCAGTTAAGCATCTGACTATCTCAACTCAGGGCATAATCTCAGGGGTCTGAGTTCAAGCCGCATTTGGGGGGGCATGGAGCCATTTTACAAAAATAATGGTAATCTGAATATGTATTATGACAGTGATTTTGTTTTTCCTTTTATTATTAGCTTTCATTAAATATTAAGCTAATTAAGCTATGATATATGCTCTGAAGGTTCAAAAATGAATAAAACATAGTCACCACCTTTATGGAGCTAGTTTGTGAGGGAAGAAAAGTATTTAAATACAAAACACTGTGAATACTATCATAGAGAAGTAAAAAGAATCATTACTTTTACCTGGTTTTCTCCTGCTATATTGGTTAATTAAAAATAGGCTAACCTTTATTAAACTGAATGAAACTTAAGTGGTTGTAATGAGTATTGAATTCCCATGGATCAATCTTTAAACATATAATATGGCTCTTGTTTTTGTGGTATAAGTCAACCTGGTGTCTCAGACAATTAAGAAGTATTTTTAAGTACTTCCATTTGCAAAAATCACTGTCCATAATTTCATGTAAAGTAGGTAGAATTCGTAGTTATTCTAAAGTACAATAAACAACAAATCTCCATTCAGTGCTTTTTCTCTTATATG

**>Fragment 12 (**Natanaelsson et al. 2006; Ding et al. 2012)

GTCGACTTTGAGAGAAGCTCTAATAACAGCAGGAAAGGAGGTCATATGGCATGGGCGGACAAATGATGAGCCAGCACATTATTGTAGCATTTGTGAGGTAACTGTTCTCGCCAACAGTTTTTTGTTGTGGGATCTCTAGTTTGGCGATGCCTTCTAAATTACAAATTTACCAGTACTTTTATATAATGCATTTTTGATTGGTTATTACAGCAATTAGTTTTAATTTCCATTTGATGGTGTAATATTTAATTTAATTCAGAAGTATTGTCTACAGAAGCATTCATGTAGTACCTTCTTACTAGATGATAATTATCTCAGATACTGAACTAGAAAAATTTAAAATAAGTATAGAGATACCAGATAACTGGATCTGTTTCAGTATAAATTCAGGCATATCATTCATAATATCATTCATTAATACAGTTGTTGGTTTTCTCCTCTCGCCAAGGCTTGCAAGAAGTACAGAAATAAAGAGGTAGTATCTCCTTTGGAGGAGCTTCCTTCCACTAGGAGAAGACAGAATGTAATGGATTCTGTCCATGTAATGGTTATGGATGCACATGAACTATGACATTTGAAGCAATGAGGAAAATAATCTAATACTGGCTTAGAGTTTATAGGCTGTTTCTAGAGTTGAGTCATAAAGTCTGTAAGAGGAGATGGTTTGTAGCTTGCAATGATCTTGAATTAAAGTTATGGAATTTCATCTTAACTCTGAGGTCGCTGAAGCCATAAA

**>Fragment 16 (**Natanaelsson et al. 2006; Ding et al. 2012)

TGACCTCTCCGATGACAAAAGTAAGTCCTCCGGTTATGTATCCATGAAGAGCGTTTAGAATCTCAAGATTGGAAATTGTTTTGTACTTTTGCAACCCTACTCATTCTTTTTGTTGTTTATGTTGTCCTAAAGTCTTAGTTATATATATAACATTCTGATACAGCATTCTGAAATGATTTTTGCTAGTTTTTGCAATTAGATTTTAGTTACTGAGGAATCTCAAAAGGTTAAATTTACAAAATAGAGAAGTTACTTTATTTCTAAAATTCTGTTTTTAGGATTCAATTTTCAGTGATCTTAATTTATTTTCAAACTTTTTTCTCTGGAAGAGCTAACACTCTACTTCTAACCTTAGAAGAGTCTATTAGAGATTTAAATGTATTTATTCGAACATAGTATTTTCAAAGACCTAGTAAGTTTTCTTCAGTATTTGCAACAAGGTGACACTTTTGATGTATTACTTTATTTCAGGTGGAAATTGCAGCTACATGAGCTTACTAAACTTCCTGCCTTTGTGCGTGTGGTATCAGCAGGAAACCTTCTAAGCCACCTTGGTCATACTGTGCTGGGCATGAACACAGTTCAGCTATACATGAAAGTTCCAGGGAGTCGAACCCCAGGTTATTTTTGTGAACTAACATATCTTGTATAACTGAAAGAAGAGTTAATCAAATATAAAACACACATACAACCCGGTTTCCCATTTGTAATTAAAAAAGCCAAAGGACTTCTTAATGCTGTCTCATATGTA

**>Fragment 20 (**Natanaelsson et al. 2006; Ding et al. 2012)

TTGTTTAGGTTATACTCGTGATTCGTAAAATACTCTCTTGGAAATACTGTAATACAAATTTTGCTTTGTATTTTAGGTTGGCTTTTTTTTTTTTTAAGAATCATTTTGAAGACTAACAAAATTTTTATTACTTATTAATAGTTGCTCTCACACAGAAAATGTTGAGTCTTGACTCTGATAGCTCTAAAATTACAACTTCACTACTACAATATCATACATCAGGTCAGAGTGATAAGCTTCTGTTTCTCACAGAAATTCATGATGCTTATTTTTATTAAATCTTCAAGAGTGGTACATAATCAAGTATCTGCCAGTTGACTAATACAGTGTATAACCGAGGTATAACTTTGCTAGTTTGTCAGTATTCATTCTACTCTCCTTTTACACATTTTCTCTTCCATTAGTTATATTTCATCCTAGTAGTATTTAATGTTTTTCAGCTTCTTTAAAAATATTCTCTGTAGTTTGTCCTACAAAGACTATGTTTAAAGATTAATCTGTCAGTCACTTAACATTCAGCCTTGATTCTTCAGTATCACTTAAATAATAGTAACATCTGTTGACCTGTTAAGAACCAAGAAAACTGCCTAAAATTATTTACCTTTTTATTTAATTGACTATTGGTATTTGTTTCAACATCTTTATTGAGTGGCTGTTCTCAAAAGTCTTAATTGTCTAGAATCATTTTTATTTTCTTTAGATATATTGATTTTACTCAGTGCAGTGAAACACAATTTAATTAACTGTTTACCAGTTTTCTTGCTTGACCAGAGACAGATTTTGATTTGTCTCATTTTTTCCTCTCCTTCTTTCCCTCCCTTCCTTCCTTTTCTTCCTTTCTTTTCTTCTATTCCTTCCCTTCCCTTTTCCTTTCTCTTTCCCTTTGCCCGACATACTGCATTTACATTTCTAATTTTTCTGTCCATTGTTTTACGATAAAATAGATGTATTTTATGAGCACTTTGCTAATTTCAGTGTACAATAATTGCTTAGCACAGCAAGAACATCCTTGTATATTAAGTACAATATTTCTGTGAAATTGAGAAAATAATTTACACTACTACTTAAAGTTCATTTTTCTTGCTTTCACACAATGGATATCCACTGGTCTTTTTTTAAATGAAATGTTGAAGTATGGTGATATGTAGATCAGTTGAAATGCTGTTGACTTATAACTGCCTTATTACAATAGTCACCAACCAGCAGTGACAACTTATGATATTTTATTAGATTTTTCTTATCTAAGAATCAGCTTAATTTTTATTTTGTTGTAAATGAAAACATCTATCTACAAAACATAAGTCAATGGCATATTTTGTTCTATGAAAGCATTACTTAAAAAAAAAAGAAAGCATTACTTAGATGAATAGATGTTTGGCCTATACGCCATAGGTTGCCATCTTCTGATTTAAAATCTTGAAAATCTGGGCAGCCCCGGTGGCTTAGCAATTTGGCACCACCTTCAGCCTGGAGCGTGATCCTGGAGACCGGTGATCGAGTCCCACCTCAGGCTCCCTGCATGGAGCCTACTTCTCCCTCTGCCTGTGTCTCTGCCTCTCTCTCCTGTGTCTCTCATGAATAAATAAATAAAATCTAAAAAAAAGTAAAAAAGTAAAATCTTGAAAATCTAAAAATTTTTAACCAGCACACATCATTGTCTCTCTTGCATTGACCCAGATTATGTAATGGGTTATAATGCTAGTATTTAGCAGTCTGTGAGTTCCAGCCAACTGTCTATTAAGGCAGATAATAGCAGCACACAGCCACTCCCTGTCTATATGTGATGTCTGTAGCCTGGGTATCACCCACTTTTATTTTTATTTCTTGGATCAGCTATAGTTTGACAAGCCAGAGTTTCTTCACATTTTCACATTTTC

**>Fragment 21 (**Natanaelsson et al. 2006; Ding et al. 2012)

**T**TATGCTTTTTGTCATCCTGCAATGTTGCCAGAAAAATATACAGAGAGATTGGGGGCAAGGGAGGGGATAGAATGCAGGAGAGGGAGGGAAAGAAAAGAAAAAAGCAAAAGTTATAAGCATGGTACAAACCTTATTTCACCCAAACACTACCAATATAAAACTAGAGAAAACTTCCCATAAAAAAAGGAAAAAAAAGCCAACTAAGATAGATAGACCATGCATTTTTTAAATAAAGTTTACAAACAATGGTTGATTTAAAGATATTAAGTATAGTTATGAAATAAACTATTAAAAGTTTTAAATATTAGAAGCAATATTCGGGATGCCTGGGTGGCTCAGCGGTTGAGCGCCTCCCTTCGGCTGAGGGCATGATCCTAGAGTCCCAAGACTGAGTCCCACATTGGGCTCCCTGCAGGGAGCCTGCTTCTCCCTCTGCCTATGTCTCTGCCTCTCTGTGCCTCTCACGAATAAATAAATGAAATCTTAAAAAAAAAATAGAAGTAATATTCTGTTCTCTGCAATATAAAATTTACCGAATATCTGAGAGGGTAACTTCAGGTGAATTAGTATAACCTAATATTACTCCAAAACTCAAACTTTTCATATATCACTGTTTTCTAACTATCTTTGACCTAAGTAATTACAAAGTAATTACAATACCAGTAAGGTAATCTGTCTTATCTGTCTTATTTTAAAGCAGTTAAACTCTCAAAATAGTTCATACTCAGAAGAGTAAGTAAGGTGCTGTATGTTTAACAGTTACAATATACCTCCTTATAACTTTAAGAATTAGTTCTTGGGGCACCTGGGTAGCTCACTCAGTTAAGTATCTGACTCTTGGTTTCAGCTCAGGTCCTGATCTCAGAGTGCTAAGATGGATCTCCAATCAGGCCCCATCCCTAAGGGCGGTGTCTGCTCCAAATAATTTCTCCCTCTGCCCCTCCCCCTCCTGTGCATGCATGTATCCCATATTCTCTAAAGTATTCTTATTCACTCTGAAGTAAATAAAATCTTAAAAAAAAGAAGAGAAAGGAAAAATATTCTTAAACATAATCGAGATTTCCCATCAAGTGTGTCCTTCTCCCTTGCTTAAGCTACCAGTTTGTTGATAAGAAACATTCAGAAAAGAGATTATTACATTATTTTCCTGACAAATTATCAATGTTTAGAAATCTGACCTCTTTTATCATGAGAAAAAATTAGCCGTGAGACAACAAAAGGGATCATTATCTTGCCAGTAAGAAAACTCTTTAAGTATTATTTAAGCTACCATAAAACACTTTCTGTGAAATCAAATATCCTGAGTGAAGACCCTCCGAAATTGATATAAAATGTATACTGATTTTAAAATAACCATACATGTGAAGTATTAGAATTTTAGCAAATTTTTTATATTTCATAAACATATCATTTGGCAGTATCATTATAAACTGAAAATAGCAGGAGACTGACTTGCTACTGATCATGGCAATTTTAGACTCACTATTAGAAATATATAAGCTTGAATTATTAATAAGGTATAAGGATGCTTAATTTTAGAATTCTTCCTATTTCTAATTATAATTAAGTCATTTTTTTTCCTGTAAAGCACACCTGAAAAAGAATGTGCATTCACACTTATCAATTTTTAAAATTAAAAAAGAATTATTTACTCCCTATCCTTTGAATTCGTACCTGTTGTTATTGAATATACTGGTTTCGTGGCCTATTTCACTTAGTGTTATTCTACATAACATAAAATGAAATATTTCAAATAATAGCCATTTAAAATGAAATTTCTAGATTTTGGAAATTTAGCATACTTTGTCCTTTTTAATAAATGTCTTCTCACAATTATTTCAAAAGAAAATCTGTTCAGAAACCTACTTTTCACAAAAGTCATTCAGAACACCCCAG

**>Fragment 24 (**Natanaelsson et al. 2006; Ding et al. 2012)

ATTCTTTAAAAAAGGATAAAATGTAAATTATTTAAAATACCTAAATGGGGAGTGCCTAGGTGGCTTAGTTGGTTAAGCATCTATCTCTTGAGTTCACCTCAGGGTGACGAGTTCAAGTCCTGCTTAATTTATTTAAATAAAAGTCAATAAAATTTTAAATTTTATTTAAGTCAATTTTTATTTAAATCAGATATTTAAGGTGTTTCATGCTGTTTTTCACATTTAATTTGATAATTGCCTCTTTTGAAATTTTCATGAATTAAGGAATGTTTGTTCTGAGTATTGTAAATTTATAAAGGAAAATGCGGGTTATCAACATTTAGGTTTTTTGTTTTATATTTTGGTTCTTGTCCTTAAGGAAATAAAAATACAAATGATGTATGAATAGAAACCTGTGTGCTTAAACCTGAATCTTATGGCATTGAACAAATTTGGTAATGATCTCCATTGAGTTCATTAATATGCAGTTGTTAGCATTTATAAGAGGAAAGTTCATTTAACAGCAAATTATAATATAGATTATAATTACTATGATTTTTGTGTATGTAAGCACATACGCACACATGTATAGTAGCTTTTTAGGTTTCTTTCTCTATACATCATAACATAAATATAGTTAACTTGAATATTAATGGAATACTTTATATATATGTATACTTTTACTAATCTCTCTCTACCTATACTCAAAATACATACAATTTTTTCCTTCCTTTCAGGTGTTACTCAAGTATTGGGAAAGTTCAGGATGCCTTTATATCTTATAGGCAATCTATTGATAAATCAGAAGCTAGTGCAGATACATGGTGTTCAATAGGGTAAGCTTTCTTTATATAAAATTAATAGTATTTTCATAGATAACAAGCTCAACAAGCATATGTAATATACTGTTTATTAGTATTATACTCAGTTTGTTTCAGACAAATTTTTTTTCTTTTCAGTGTGCTGTATCAGCAGCAAAATCAACCTATGGATGCTTTACAGGCCTATATATGTGCTGTACAATTGGATCATGGGCATGCTGCAGCCTGGATGGACCTAGGCACTCTCTATGAATCCTGCAACCAACCTCAGGATGCCATTAAATGCTACTTAAATGCAACTAGAAGCAAAAGTTGTAGTAATACCTCTACACTTGCAGGAAGAATTAAATATTTACAGGTAAAAATTTGAAATAGCTTATTCTCAAGAACTTTTCCATATGATAACTGTCATGAGGAGGGTGGCTGGAAATTGTGTCTTTTTTGTTTTCATGTCAGTAACTTTTTTCCATAGTTTTTAATATTTTGTGATATTTTACTTTTCATTTAAAGTAAAAGACGTAGTATTTAGTGTTCTTTTTTTGCACATTTACTTTGTTGAGATACATTAATTTGCATGATTTTTCTGTGTGCTATCTACATTTTTAAGTTACAGCATTTTAAAGGAAACATTGTTTTCCACTCTTGACTGAAATTTCATAAGATCTTTAAGCTTTCTGAAAAACAGAAATTACCTTCGTTGTGACTCACATGAGTATATTCTAGTTGCCTTCTTTGTATTCAGGATATATCAAAACCCCCGTATTTAGACTCTATTTTCTTTGAAAAATAAATACCTGTAACATTGAGGAAGATTTAGTGGACTTGCTCTTACTCAAGTAGGCCTGTGTTAAATTTGCTTTTTACATAATTTTTCCTAGGCTCAGTTGTGTAACCTTCCACAAGGTAGTCTACAGAATAAAACTAAATTACTTCCTAGTATTGAGGAGGCGTGGAGCCTACCAATACCCGCAGAGCTTACCTCCAGGCAGGGTGCCATGAACACAGCACAGCAGG

**>Fragment 27 (**Natanaelsson et al. 2006; Ding et al. 2012)

CTGAAACTGTTATTTTAGATAAATGAATATATCATAGTTTGTTTTTTTAAACATAATTTCTATTACTTATAAAAATCAATGTATTATTTCAGTTTACTAATTCTGAACAGTAGTCAACCATTCCCATTTTGTGACAGACTTTCCCATACTAACCTGCATGCCTTCAGAACTTCCGCAGAACTTGAGCATATGGACACAGACATTGATGGTATAATATGAGTACCAGGTTTTTGGCTAATTATAGCCAGCTCTGCTTTTATAGAGCTCTTTGAGTTCTCTAGACTTTCTCCATTAACCGTATATAACCTACTGTGAGGACTGTTAAGGCTGGGAACACTGTGTGTGGCTGTTTGTTCAGTGGATTTTGGAGAAGGTGTTGCTGTAGAAATGACTGAAGAGGGTGAAGAGGCAACAGAATTATTAGTCTTTGTACAAATAGTTGGGTGAAAATGATTGACTTTGTCACAGGCTTCCCTACCCCCAATGTCATTGGCTTTTCCAATCAACAAGGCAGAGATCTGAGGATTGTCTGAAATCTGTACATTTGGTGAAGTGGAATCTCCATGTTTAGGACTGCAAACAGGTTCTGCCACCAACTGCTGAACATGATTAGAAAGTCCTTTAACACCAGCACAGCCATTAGGTGTATCTTCAACATGCCTGCTTGTTTCAGGCACCAAGGATCGATTTCCTGAAAGTTTGCTCTCTTTGGTAAAGGTAATGCCTTGTTGTCCACCTGAGGTAGGAATATGAGAGGAGAGGTGATTGAGAGCAGCTCCCTGTGTTACTGAATGGCTAGGCAGAGTTCGAAGTCCAGCCTGTTTCTGAATAGCAGTGCTAGCTGCCTGGAGATACTGGGCAGGCCCAGTGGAAAAGGAAGGATGTTCATCATTAGGTCCTGCCAAATGTGAACTCTGACCTTTGTGAAGCCCCTTAAAAAAAAAAGAAAGAAAGAAAGAAAGAAAGAAAGAAAAAGAAAGAAATTACCAAGTTTTGTTTTAGTGTATTATTATACAAACTCCGAATACATATACAGAAGGACACGGAGTATTAAAAATGTTTAATGATCTATGAAAAAGCAATTAAATGTGAAACATATTTTAAACCAAAACCAGAACTTATTTTCAAAAGTTCCCATAATCCAATATTAACATGGTTAGTATATTCAGAAAAATTATGCTGAGCTGTGCTACACAATTTGATTCTACATATTAGTATGTCAAAATATTTAATATAAGCCTTTAAGAATTTTTAATGTTCAATTCTGATACATACATTTTCACCTTCTGAAAAATATATTTATAAATTTTGATATGGGGGAAGATACATACATAGTCATTACCAAGTGAAACCATGAAACAACCCAAATATAAATAGCACAAGTCACTTATTTAAAAACTATTTATTAATTTAATAACTTAAATGGATTTCCATATTCTCACTCCAACCCCTCATAACTGACAGACATTTTAAGTGCAGTTCCCCACTAATTACTCAAGAATTGGCAGTTTTACCAAAATTCACATGGATTTGAAGAATATATTAAGGTTAGAGCATTTTTATGGGATAGTTTATTGAAATCATTAAGTTTTGAACCAAAAGGAAGTTATTTCATGGAATTAAAATATGCAATGAATTAAACTAATTTTACCTGAAATTGTCCTTGGCATGGTAAACTGTTCTAAAAAGATTATCATTTTATTTTATGAAAGTAATTAAGCAACATTTATTAAGTAATATGGAATAAGGAAACAATAATACGTTGCAAGCTAACATAACACTAGAATGTGAAAACTATTAACTTCCCTGATGTTAGAAATATAATTAGACATTAATACATCTTTAATATAAAACTCTATATGGAATTAAACCTCAAAACTGTAGAGTCTATCTTAGAATTGCTTCTTTCTGAATTGGTAACTAATTTAGCAACCAAGCAAAAGCGTTTGCTGTCATAGAAATAGAAATAAGTTATATATACA

**>Fragment 28 (**Natanaelsson et al. 2006; Ding et al. 2012)

GAACAGAAGTGTTGAAGAATTGGGAGTGATCACTCAGTCAAGTTCATTTATTTCCTCATTCTATACACAGGGGATATGAGTCAAGAGAAGTAAAGTGGTTTCCTTAAGGGTACTGAGTGTCACAGAAAGGGCTGGAACTCAAGTTTATTTGGTGCTGCCTGTATTAGAAGCCAGCCTGCCTTAAAGGAATCTACATAAATTGTTTTGGGGGAAAAGTTCTTGGTGATTGGACTTAGATATTAGTATTCGGGAACATTGACATTATGTTTTGACTTTTAAAATGGTATGTGATAATGCACTTTTCAGAAAATTTTTCTTATTTTCCTAGCATTTAGGCAAGTGTGTCTGATAATCTGAAATTTGGTTTTGTTAAAATTTTTTTCTGTTGCTATTCACATCAACTCTGTGTCCTTGTTTCCAGTTATTACTGACTCTTTTTTATATTCCTTCCAACGTGGGTTTTGTGTGCTCTCTAAAATTGCTTTCTCTGTTTTCTTTTACTGGTGTTTCTTCTATCTCTTAACCCACAAATTAGTCATTCTTTAGATTTGTTTCTCCCTCACCTCTTATATCCATCTTGTGTTCTTAAGCCTCTCTTTCATATGGATAAATTGCAAATTAAGATTTCTAACTCTTAAGGTTCTGAACTTGTTTCATAATTCAGTTCTGTTCACACCAAAACTCTAAATCAGCATCTTTTAAAAGCTCGTATATCGTGTTCTTTCTCAAAATAAATACTTTATTCTTTCTCACAGTAAGTTACCTTTTTATATGTTTTACAATTAAATCTCCTTTAGTATCTAGTGAAGTCATTGAGTCTTTATCAATCATTTATTATTATTCTTGTATATAATTTTCATTATTTCCACTATAATTGTGGCTCAAGAGTTTGTATCAGCCTTGTGATTTTCTGTCTCAAATTTATTTTCATTGAGTCTTTAATTTTTCACTTTACACATTGTTAGAAGATTGTTTCTGTACATTGGACCCTTGCCTTTTTTGAAGGATAGATTCAAGTTGATCACAGATTTAAGTTTTCCATTTGTAGAGTAAGTTTTTAGATGATATGCTTTTTGTCCTCTTTCCTACTATAATCAGTGGTTCCCAACCATTTGGGTTACCTGAATTAGCAAAATTTGAATAAAAAATAGTAGTGGTCACCTTAAAGTGGCTATGGAATCATGTGGTTCCTGAGATCGAGCCCCATATCAGGCTCCCTGCCTAGCGGGAACCTGCTTCTCTCCCTCTCTCTTCCTTCTGCTTGTGTTCCCTCTCATGAGCTCTCTCTCTCTCTGAAATAAGTAAATAAATAAAATGTTTAAAAAACAATTTTGCTATTTTGTATCCTCCAATTAGAACTGTATATACAAAGAATTCCATTGAAATACATATTGAAATAATTCCTGTCTTAAGAAAATAAATTTAAAACCCAGACTACTTGTTGGCTGTGATTTGGGTAGTAAAGTTTTCCTTTCTTTAAATCATTGTAAGTTTTGTGTCTTTGTTGGTTAGATGGTTTTTTGGTAAAGTAGCCATATACTGTCCTTTACTTTTAACATTTCTTAATATAACTGCTTTTTACAAAGTAGGATTAGGGATGTCTGAGTGACTCAGTGGTTGAGTGTCTGTCTGCCTTTGGCTCAGGGTGTGATCCTGGGGTTCTGGGATCAAGTTTCTGCATCAGGCTCCCTGTAGGGAGGAGACTGAGAGGCCTATGTCTCTGCCTCTGTGTCTATCATGAATAAACAAAATTCTTTAAAAAAGGATTAAAATGTAAATTATTTAAAATACCTAAATGGGGAGTGCCTAGGGGCTTAGTGGTTAAGCATCTATCTCTTGAGTTCACCTCAGGGTGACGAGTTCAAGT

**>Fragment 29 (**Natanaelsson et al. 2006; Ding et al. 2012)

ATAGATTCCCCCCTCCTACATATCTGAATTTAGCTTTTTAAAAAATAGCTTTACTAAGGTATGATTCACATGTCATACTTTATAATTTCTTCTATAGTCAAAGTGTAACCAAAAAAAGAAAACACAGTGTAGTTTTATTTATTTTTTATTTTTTTCACAATGTAGTTTTAAAATGTACAGTTCATTGATTTTTAGTATATTCCCAGATATGTGCTACTATCACCACAGTCAATTTTCCAGCCATTTTAGCACTTCAAAAAGAAATCTAATATCAGGTTTTTAATGTAGTGGTTTTATTCTGTTTATACTAAACCATAAAATCCAATCCTACATTAAATAATAAACTATCTTTTTCCTTTCTTGCATTTACTTATAAATGTCCTTATTAGACACTACATAAGGGATGCCTGGGTGGCACAGTGGTTAGGCTCAGGGTGCTTTTGGCTCAGGGTGTGATCTTGGGATCATCAGGCTTCCTGTGAGGATTTTGCTTCTCCCTNNNNNNNNNNNNNNNNNNNNNNNNNNNNNNNNNNNNNNNNNATAAATAAATAAATAAATACTTTTTAAAAAGACCCTAAATAAAACCTGGATAAAGTAAATGCTTGAGTAAATATATGAAATATACTGTAATATTTTTATTATATAACATTTGAAATTTGAACACTAATATGTTGAATTTTAAAAAGATATTCCTAATTTGAGTTTGAAATAAAATAGAAAATAAATGTCTGTTAAGTACTTTTGAACTACCTTGACTATTTTATTTTAGTATAAGTTATGGATATCCAGGTGGAAAATAATCATGCTTAAAGATTGTAAATCTATTCATGTAACTAGTAGTTAAATATTCTGTATTAGCTGTATCTTAACCTTGCTTAGACAGGAGTCACTTAGATTATTTTATTAGGATTATGAGTCAATCATTAAATAGTGTCACTGACAAAGTTATATGATACAGAGCACATTTTAAAATTTTTATGTGATTTGTACTTGCAGCCACCTGGTGAGTAATGGAAATAATATTTAATGTAAACTTATGAAAATTAAATTGGTAT

**>Fragment 30 (**Natanaelsson et al. 2006; Ding et al. 2012)

CTTGAAAGAAGTGCCAGAAGAGAGAAAAAGGTAAAACTCCAATTCAGAAACACATACACTTACACAGTAGATGTATACAGTATCTAGTCTTCCTTCCTTCTACCCCTCGCCAATGTTATTTCACCTCTGAAATAATACCTGAAACTCTGCAAATTTTTATTTGCATATGTAAAAATGATTATAAACTCAGCAGTGCTGCCAGGTAGGAAAATAAAATGTTAACAAGAGGTAGCATTCATAAAACACGGAATTAAAGAAGTTACACAAGGTTAGGCCACTTAAATCTAGTGACACTGAAGTCCAGTGCCAAGAGCCTTTATCATCCATCTCTTTTCACAATGAGGGAAAAACTGGGCTCAGAAAGGCAAATTTTACCTATGTTCTCCTGCAATCACAGGTACAGAATTACTTGCCCAGATAGTGGAATCGTAAAAACTGACACTGCTATTAATATTATCTCACATAATGGATAACCAAATAATCAATGAAATGGTCATTCCATGTCATGTTTTCTCCATTACCCTGAAATTTTAGTCCTTTCCATTTTCAGAAAGCTAAGGAACAAAAGAATAAAGGACAATGTAAAGGTATAAGGACTTACAGGTAAAAAAGTCAGTAAGAATCTAATTTTTAAAAAAAGATTTTATTTATTTATTCATGAGAGACANNNNNNNNNNGGCAGAGACATAGGCAGAGGGAGAAGGAGGTTCCGTGCAGGGAGCCCGACATGGAACTCGATCATGGGTCTCCAGGATCACACACCCTGAGCCGAAAGCGGCACTAAACCGCTGAGCCACCCGGGCCACCCCAAGAATCTAATTTTAAAAGCAACAAACATAAATAGAAAATAAAGATACTTAGGAAAAGCATCTTAAGGAAAAAATAACGTTCAAGACTTAACAAACTATGCCTTACCTCTCTCTAGATCTTAATTAATCTGAAGAGAACTTTTCAATATACAGATGGCTTAAAAGTATCAGCAGATAATTTTTGCTTGATTTTGGGAATCC

**>Fragment 31 (**Natanaelsson et al. 2006; Ding et al. 2012)

AGCAGGACCTATATGAAATATGCTCTGGAAAAAAAATAACCCACTAAGAATATAGTAGGTTTAAGAGAATGAGATGTATTTAAGTATGTATACATGAGTGGTATACACATTCCAGTCAGAGTGTCAGCAACTAAAAGCATAATTAAGATAGAGGTTTGAAACTCAAAAGATCTGAAAGAAGCTGGAATAAAAGACAGTGCTGACTCCCAATGCCTATTTTGTCAAATGGATTAACTATTAATGGGATTCTTATCTAGTCCACTTCTTATATCTTTAAACTGTAGACACATTTATGATTTTTTTAAGTAGTTCATAAGATTCTTAAATATATATTTGTCATATCTCCACATAAAGATAATACTATATAATATTTCATTTGGAGGGACCACAGTTGCTTCCAAATTATGGCAGTTATGAATGAGGCTATGTAATTCCTTTGGCCTTTCATAAATGCCCTTCTTTATCAGTTTGAGATAATTTCCCTGTATTCCTAATTCTTTTATCGTTTTTATCATGAAACGATACTGAATTCTATGAAATGGTTTTGAATTTTGTCAGAAGATGTTGAATTCTTACATTTTTTTTTTTCCTGCATCAATAGAACTGACCATATATTCCGTTTTAGTTCATTCCTTTACCCTAGTCTCTTGGAACCTTCATTTTACCCTATATATCTTTGAACTTTTGTATTCTAAATACTTCCTACAAACAGAATCATGCAGTATTTGTCCTTTTGTGCCTTACTTGTTTCATTTACATTACCATCTGTGTCATCTCTTGTCTAGCAGGTATCCAAAA

**>Fragment B (**Natanaelsson et al. 2006; Ding et al. 2012)

CTGATAGGAAGCATGTTGTCTTGCACTTGATGCACTGACGCTCATCATCTGGAAGCAGCTCAAAAGCCTCTCGTTCAGCCTCCGTGATGCCCTGGGGGTGTTCAACCCACATATACTGTAAAGATTAGGAGCAGGGTTGTGAACACCCCACCTCAGCCAACACTGAGCCCTGCTGTTGGCTTAACCTTGTAAGGGTAGCTGTAGAGAAATCGGAAGAAAAAAATATAAACACATACAGTTTCTGTCTTTCCACTTCAAAATGTATGAGTCAGAGATGAGGCACAGAATGACTACTCAATTTTTATCTGTCTCCCTTCCACACATCTGTTATTTTTTTTTTTTTCACACATCTGTTTTGAATCCCTAATTTGTACTGCCCACCACCCACCTTCTCCAAAAGAGCTTTTCGTAAACGTCGCTCCTCTTGCACCATAATGAACATCTCCTTGTGCACAGCCACAGCCAAATTTAGGTCCAACTTCTCTGGAAATGCAGCCATCTTGCAGATGAGCTCTTCATGGGAAAAAACACAGTAGCGCCGGAGCCGGCGGTAGTGTTCAATGCACTGGCGTCCAGCAGGTAGCTGTAAGTAGTTCAAGACTGTGTATGGCCATCTGCAGCCCACAGTTTTTAGCTTCTCCAAAATGACCATATTCTGTCCACATTCTTTATCCCTTTCTATCTATAAGCCTACCACACTCCAGACTCACCCAGTCAGCAGTGCAAAAGTTGACAGCCTCAGCAAAGTTGTAGCCTTGATTAAAGCCACTGTGGTAAGCACGAGGAAAT

**>Fragment G (**Natanaelsson et al. 2006; Ding et al. 2012)

CCTGAGCCAAAGTCCCACGCTCTACCCCTGAGGCCCCCGGGTGGACCTTCACTCTNNNNNNNNNNNNNNNNNNNNNNNNNNNNNNNNNNNNNNNNNNNNNNNNNNNNNNNNNNNNNNNNNNNNNNNNNNTCACAAAAATAAAGTAGTGGGAAATACGGATAAAACTGTGCTATAGATAAAAATGTACTTCCATGGAAAGCATTCTTTGAAGCTTTCGTTTGAAGGAATCCCTTTGCAAAGGACAGGTTCATTGGTCTCTCAGCTACTTTGCCTGTCTTTGTAATCCCCAGATAATTAAGGACTACATATGTAAATTATATCTTCTTTGGTACTGTATATTATCTCTCCTTCCTGCTGTGGAAATCCGGTTTTGTCCATATCTGCAGTTTAATTCCTCTCCTCTACAGGTATTTGCGCACTTTCCCTTCCACTTTAAACCCATCCAGTAACATCCATCCAGTGCAAGAAAATCTTTCTTTTTTTTTTTCTTTTAAGATTGTATTTATTTATCCATNNNNNNNNNNNNNNNNNNNNNNNNNNNNNNNNNNNNNNNNNCATCCGCACAGGGAGAAGCAGGCTCCATGCAGGAAGCCACCCGGGAGACTCTGGGATCACAGCCCTGTGCCAAAGGCAGACACTCAACCGCTGAGCCACCCAGGAGTCCCGAGAAAATCTTTATGTAACTCGTGTTCATTTTTATATCTGTAGGACACCCGGACTTTTGCCTCTTTTACTGAAAATTATCTTCTAACAATGTAATTGCTTCCATTGCTGACTGACTTACACACGCACAAAGGTAGAGGAAAAAAATATTGAGAATATGAACTATTCAGATTATGGGGCAGTGGGATAGGAGGTGGGAGAGCCAAGCCTGGCTAATCTAATGTAGATGTCAATGAAAACTTAAATCAAAGGCATTACCAAGTTTATCATACAGGCTAAGGTAGAAAACAGAGTTTGCAAAGAAAATCATCACTTTTTAAAATAAAGATAAATTGATGGTCAAAGTACATACCGTCTATCAAAACCAACTTTCCAATACACATATTCCAAAGTTTTTCAAATACTAAAGCTCAAACACTAAGATAGTACATTTTATTTTTTATTTTATTTTATTTTTTTAAATAGTACATTTTAGAGCTCAATGCCATGTTTT

**>Fragment K (**Natanaelsson et al. 2006; Ding et al. 2012)

TATATATCCCAAAGTATATCCCCCCATGTGTACAAAGGCATAGATGTGCTATGTGCCAAAATATTTGTTTTTGATATTCACTTATTAATTTGAATACTCTCTCATACTAACAAGATTTTCCCAAGGAGACACAATATTCCAGTATTTAGTGAATTATTTTCTAATAGAATAATATCAATTAAAAATAATCCTTTAAAATGTAAAATCTGTTAATGGGTTATTAAAATACCTGTAAGTGGACCAACATTCCAAGCAATATTGTTGCACCAGCCAACAGCCTGAACCCAATGCACAGTGCCTGCATTTATCCAGACCAAATCTCCAGGTCTTTGAATAAATCTATATACGGGGACATTTGCTTCATAAAGATCTTCAAGGTTGGGCCACCAAGAACTCATCAGGAAATTCAAATTATTTCTGGGGGGAAGGGGGGTAAAGGGGGGGAAGGAGTAAATAACATAATGAATTAAGTTTTTTAAAATGTCCCATAAAAGATGAGACAATTTCCAAATGTTAAAAGATACTTTCATTATGCATTTCATTAAAACGTAATAGTACTGTAATACTGTACTATAACAAGAACTGCATCTTTCAAAAGTGTAAATATTAAGGGGTAATTCTGAAAACAACATTAATAGCAACTGATTATGTAGACTAATTCAAAGATCTACTCTGTATGTTTATTATTTTCAGAGTTATGTTCCAAACTACACTTAAAAATAGACTAATTTTTTAATTTTCCTTAAAAAAACATAATGGTTTACACTCCTCCTTCCCGTAGAACCTCTTAATGTCAAGGAATCTCTAAAAAGGAAATTGAGAATAGAGATTTAAGAACCCACTTAACTTCTTAACCAGAAGCACTAGGCAAATATAAGAACATTAGACAGATTTCC

**>Fragment N (**Natanaelsson et al. 2006; Ding et al. 2012)

**T**TGAACTACAGAAAAATGGGGTAATGATTCCAAACCCTGTGGAAATTGGTCTTAATGAAATAATAAGAGCACATCATGTAGCAGGCTGTTGAACTTTCCGGGGATTTGGTGTTGCTGTCTGACTCACTTTGTCGGTATTGATAGCCATGAGCTAAGAATAGCATTAAGGATTAATGTACCAACAATTAATATACCTTATTTTTAAATTTTAGGCTCCTTCATTGTCATCATCCTCTTGATATTATGCCATGAATATTAAGTGGCACCTTTTCTGGTCTTCAGGAAAGGTCCTGTAGCTGTTCATAAAACTGCTGACTAAAAGCTATGTACTATGCAACCTTCCAAGAATAGAGTGTCAACTAACTGGACATAGGAGAAAGCTGCCTCTACTCCAGGACTCTTCACAAAGTTGATCCAGCTGTAACTAATAATAGTAATAATCACATGTTTTATGGTATCTGACAGAATGTGGCAGTGTCACAGACTACTGACTTGAAGATGATCTTTTTCTATGTTCCTGTATTTCTGAACAGCTAAATTACTTTTCTTCTATATTTTACCTTTTCATAATTTTTCTGAGGAAAAAATACTATTTTAGCTGGCCATTTGCTTGGTAATAAGATAGTTAACAAATTCAAACCTTTAATCATCTGGGTTTTTTTTAATGTGAAAAATTAAATGAGAAAGTTTTTTGCGGTTATGTTTGGTTTGATTTGGTTTCTTACCATTTTATATTTTTCTCTGTTTGAGGCCACAGTGATTACAGTTCTGGTTCCAAAATAAAACTTTAAGTAAAAACTTATACCCTAATAGCCAAGACACAAAGATAATGGATTGCACATAGAGTAAGGAATAAACTTCAGATTTGTGATTTTTGTTTCTAATCCTGATACAGATTTACACTATTTATGAATACATATTTATTGCTTGAAGATATTTGTGAATGGAATGTTGTTATTTTATCTGGGGTTTACCTGCCATTAAATATTAAGGAGTTCTGTAATTTTAAACACTACTCCTTTTACATTTTCTATGTGTAAATAAAACTGCTTAGCATTGTACAGAAACTTTTATTAAAATTGTTTAATGTTTAAAGGGTTTTCCACTGTTTGAGTTTTAAAAAGGACTTTCTGTAAAGACATAGCGTTTGTTCATTTTCAAATCTGATTATATGTATTTTATATATATACATATATATTCACTCGTATAGTGTATAACATAGAAATAAAAATATATAATTGTATAAAGATAAAACAGATTTTAAAATTTTCACATTTA

**>Fragment Q (**Natanaelsson et al. 2006; Ding et al. 2012)

AGAATCTTCTAGTGTTTTATGGGTTTAAGAATTGATATTAGGTGATAATAGATCATTTGTTACTTTGGGTAGCTTTATAACTTAAAAAACTTTGTTTCCTCCTAGCCTGCCAGTATAAATTGGCAGTGGAACGGTATGAATGGAACAAATTGCAAAGTGTGAAGTCAGTAGTACCCATGGTGCATCTTTCTTGGAATATGGCACGAAATATCAAAGTCTCAGATCCCAAGCTTTTTGAAATGATTAAGTAAGTGTTTCCAAAATCACTGTAGTCCCTTTTTTTGTGGGGTGTGGGGATGTGGTGGGAATGTTAACTAATGAACATTTTTCCCCCAAATTATCTTAATTAACTTCAAATGATAAAATGGTCCAGAAGTCTATACAAAAGAGAATTAACATGTTAAAAGTATTTTCTTCCTTTACAAATAAACAATGATTATTATGGTAAAGAGAGGAATAGAGGCATATATACATAGTTCCATGCCAATAAATTCATGATCTGTGCTTTCTCTTGCCCTCTCACCAAATTGATTTTCCAGAACAACTTTAGTTTTAATATTGGAAGTCTGCTGTAGGAACAGAAAGTGATAATTCTATTATAAAACAAATGTTCAGTAAAGAAATTAGCATTTAAAGTGGTGATTTAATTATAATTATTGAATTTTCCAACTATTCTATCTAAATCATCACCTTTTTAGCTTAAGTTCAATTTTAATTTAATAGATTATCAGTTTNNNNNNNNNNNNNNNNNNNNNNNNNNNNNNNNNNNNNNNNNNNNNNNNNNNNNNNNNNNNNNNNNNNNNNNNNNNNNNNNNAGTAACTCATAGTTACTCTCATAGTAACTTCTCATTTTTTAACTTGATTTCCATTCTATTTCTTTCTTTTGGGGTATATTTGTAATAATATGTTTTCACAGACTGCCAAATAACTGTATTTCTCCAAACAGCAGTTTATATGCTTTTCTTTCCCTCTCCCCTTCATTATCACCAATGAAAAGTAACTCTGGTCTGAGAAATGTGGGATGTTACAGATGATACAATTAAGCTGCTGGTTTTTTTCATTGTTACTGTTTTTGAGATATGTGTGTGNNNNNNNNNNCACACAGGAGAGGAAGATTTTTCATGTCTTTGGAATCTGTTTGGCCAGTATCTTTAAAATCACTGCTAGTTTATTACAATTTCAGTTCTATATTGAAAATTTTGTTTAATAAATCTATTTAAACAAAAGCTGAGGCTATAGTAATATGCATATCCACTGTTTTATATAGAGACTCATTCCATTTTTTACATATATCTGAAATTTAAAATAAACATTTGAAAGAGCATATAGTCAAAACTCAGTAATTCTTATTAAAAATGTACACGTTGTCAGTAATTTCTCTATCAAGCTCATTACTTTAAGTATGTTTCTTGAAAATATTCACTGGCTGGTTCAAAATGAACCATTTGTTCTCAGTGCCTTGGATTTTTTTTAGGATTTATGCAGAATGCATCACTTCTGTTTGAGAATTGCGGAGTTTATGTCAGACACTGTTCTTGGCAATGAAGATAGTGTGTAAGACAAAATCTCTTTGAGTTCATATGGTTTAAATTTTAGTGGAGCCAGAGAGGCATATTTTCAGTCAGGTAAATAACTTACCAGTAGTAAGTAAGGTAAGTAAAATAAAATAGGGAAATTTTTTGTTTACAGGATAGATAATACTGCTTTTCCTGAAATGACATCTAAGGGTATGATTTGTGTAGTCATACCTGAATAGTGAAAAGGAGATATTCTTGAGAAGATTTAGGAGTTGAGTAGAACATTTCCTATGAAACATGTGAAAGGCTTAAATTAAGA

**>Fragment R (**Natanaelsson et al. 2006; Ding et al. 2012)

AGTACTAATTTAGAGATTCTTGAGGATATGATGAGAAATAAGAGATGCTTTCTCTTTAGTTAAGAAAATATATGTGATTACTGCAAGAATATATACTAAATTTGATTTCTAACAAAGAATATGTGCTTCTTTAAAGAACCTTGAAATGAATTACTCAGACTTACATGAAATTTGCACATCTAGCTGTGGTAAGGATCCACTGTTCATTCAGTATAGAGCCAGCACAGAAATGGGAGAAAGAGAGTTGCACAGAAACCATCCAGGGAAATTCACCCACTTCTGCTTCCCAACAGTTTGGACAGTGGGGAGCAAGGCCAGGTCGTAATCCACACTCTGTAGAAGGAATCCATGTTTCCAATAGGTTGAAGTTAGTATTACTACCTCCTTTACTTGATCATTTTGATTTCCTATTTTTAGATTTTACAAAAGTGCTTGTTTATTTTCAACTTTAGGAAAATGCTTGGGGAAAAAAAGGAAAATGCTTGGAACTATATAATTTTAACCCTATAGAGTTGGCTATGGGAACAGAAATTGTCTGCATACACTCTGAAAATACTATATTCATTACTCAAATAGGAAAAATAAGCAGTTTCTAAGTACTACACATATTTTTATAACATGCTCTGAAAGAAACTATATTTATAAAACAAACTGTTTCTAGCAAAAATTAATCTTCTCTCAATAAGTACACACCTTAATATCTTGTTATTTTTGTTCCCCCTTCCCTGATCTAAATGTTTTGAATATTATCCTGCCCAAGTATGAGTAAAAGACTTCTGACTTAATCAAAAACTCTTGTTTTAGGCTCTAACAAAGACCTTGCTCCCAAGGATAACTCAAATAGATGACTACACTGAACTCACCTGTGGTTTCATGAGAAATAACAGCTGTCTTGAAGAGCCAGAAATCCTGGATAGTCTCTTTCCAGATCACACCCAGGTGACACTCAGCCAAAGAAAT

**>Fragment DBY4** (MG594247; Hellborg and Ellegren 2003, Fain and Straughan, unpublished)

TGGCAGCCGTGGTGAYAGRACTGGCTTTGGCAAATATGAACGCAGTGGACACAGTCGTTGGTGTGACAAATCAGATGAAGATGATTGGTCAAAACCACTTCCACCAAGTGAACGATTGGAGCAGTAAGTTTTTGAAATGTGTGTTGACTGATGTAAAACTTACTACTTAGTATAACATGTAATAATCATTTGATTTTTAGGGAACTCTTTTCTGGAGGAAACACTGGAATAAACTTTGAGAAATATGATGATATACCAGTAGAGGCAACCG

**>Fragment DBY5** (MG594244; Hellborg and Ellegren 2003, Fain and Straughan, unpublished)

CTTTGAGAAATATGATGATATACCAGTAGAGGCAACTGGCAATAACTGTCCTCCACATATTGAAAGTGTAAGTTGCTTTTGTTTGACGTTTTAACAGTTCTTTGTTACCACTTAAAAACTTCATTAGATTATAAAGAAAGGAGAGTCTGAGACTTTATATTACCATTATGACCAGTGCTGAAACAAAATGTCAAACATAAGGAATCCAAAATTAATCTAATATTAGACCCAGGAGTCTTATTTTCTTAAAAACACGATGATACTTTTAACCTCAAATTATAGCAAATTTGGTTTGTAGTCACAAACCACAAAAGTAGATATTTGGGGGTGGTTTTATTGTCATATTAGTACAAAACAGAAATTGTAACAGCTAAGGCATTTCTAGTTTTATGTGTATTTTACTTAATATTTGATAGTTCAGCGATGTTGAGATGGGAGAAATTATCATGGGGAACATTGAACTTACTCG

**>Fragment DBY7** (MG594246; Hellborg and Ellegren 2003, Fain and Straughan, unpublished)

GGTCCAGGAGAGGCTTTGAAGGCTGTGAAGGTAAAGATTGATTTCCCTCTAAAATAAGTTGTTGGCATAGAAAAAGCTTCATAAATATAAGTTGAGACCTTCCTTTTAAACAATGGCAAATATGTTTTATTTCAGTTCCTTTGAGTGTTTTATTATATCTCATGAAAATGTATTTTTGATGTAATTTTTAATTATACAGGGAATTTTGCTGTAGAGTTGTTTTAAGAGAAAAAAAGAATTCTGGGTCTTTGAGTTTAATTTGTAAATTATACTCATTTCTTAGGAAAATGGAAGATACGGACGCCGCAAACAATATCCTATCTCCCTAGTTCTAGCCCCAACAAGAGAATTGGCTG

**>Fragment DBY8** (MG594245; Hellborg and Ellegren 2003, Fain and Straughan, unpublished)

**C**CCCAACAAGAGAATTGGCTGTACAGATTTATGAGGAAGCCAGAAAAGTAAGTGTGCATTTTACTGATTATTGCCATTCTCATTGATTGTAGGGATGTTTTTATGAAAATCTGATTAAATTATTTGCTTATAGTTTTCATACCGGTCTAGAGTTCGTCCTTGTGTAGTCTATGGTGGTGCTG

**>Fragment SMCY5** (MG594248; Hellborg and Ellegren 2003, Fain and Straughan, unpublished)

**A**AGGATAAGACTTTACGGAAGAAAGGTGAAACCTCATAGGCCAAGAGGCACTGTGTGGGTTAAAGTACAAAAGTGGAAGAGGTGGTTTGTTACTTTGATAGGAGAATTGATTTGTTTTCAGATTTGTTTCTGACTGCCCTAGATAAAGAAGGGGTTTTGTGCCCCCGAGCTGTTCTGATGAAAGAGGAACCAAGAGAGGATGAGAGAGTGATGTCCTCATTGCCCAGAGAGGACTTGAATCAC

**>Fragment SMCY9** (MG594249; Hellborg and Ellegren 2003, Fain and Straughan, unpublished)

**C**AAAGTACGAGTRGCYTTGGAAGTGGAGGATGGCCGGAAGCGCAGTAAGTGATGAGAAATGGAAGGATCCATGGGGAAGCTCTGTTTGTTTTTTTCAGCACTCCAAGCCTCCCTCCAATATTTTTCCTAATATTGTATACTTTTTGCAACTTTACCTCTATCTTCAGGCSTTTGAAGAGCTAAGAGCA

**>Fragment SMCY11** (MG594251; Hellborg and Ellegren 2003, Fain and Straughan, unpublished)

TGCCATGCATCAAATAGAGGATGTCAAGGTGAAGAGGAAATTCTTATTAAAAACAGATACTAGCAAAATCTGAGGTAGCAGGGAAGCTTGAGAACCTAATTGTTCTAAGTAATTTCTTATAATACATCTTTCTTACAATTTATAGAATTGAGTACTATTTGTATCATTGAGTACTATTTGTATCATAATATTCATAGTATATAGCTTCATAACAGTATAGCTTATAGCTTATTATTATTATTCTTATAGCTTATAGCTTGTATACTTAAAGCGGGTATCTTTCCATGCAAAAATAATTTAAAATACTGAACACAGGGCTACATACCTATCTGGCTTTTGGCACTGGAAAATGTGAAGGAGATAGAGTTGTTTTTTGTTGTTGTTGTTGTTTTTTTAATAATTTAGAGTCTTAGGAAGCCTTCGAGAAGAATAAGATGGTGTTAAAGTGGGCCTCAAAGGATAAATTTCAGATAACTAAAGGAAAAGTTGGGTTATTATGGCAGATAAAGGAGTTTTGTTGATATTAAGTAGATCGTGTCTTATTATATGATTGAAGACAAGCGTAAACACCTAAGAGAAAACATAAACAGATTAAGGGCAGGGGTTGTGTAACTAGGTGGCTAAAGAGTCTATCCTTTATCCTATATCTCTGCAGGACGTCCTGGAACAGGTGG

**>Fragment SMCY17** (MG594254; Hellborg and Ellegren 2003, Fain and Straughan, unpublished)

TTTTTGCTCGAGTAGTCGGCAGTATCTGCGGTGAGCCATGGGGTCTACTTGGAGGATACAGTGGAGGTTACAGAGCTGGGCCAGATGTGCTTTTCCTTGCCCCCTTCCTCCAGGTATCGGTACACCTTGGATGAGCTTCCTGCCATGCTGCATAAGCTA

**>Fragment SRY** (AF107021, Meyers-Wallen et al. 1999)

CCCGGTTAGATGTTGACCTTATTTGGGAGTTTTGCTTGAGAATCGGTAGGTTGGGCTTCGGGTGATGGTCAGCGGAGCGGGTTTGAGGCAAGGTGCTGGGCGGAGAAATGAGTATTTTAGAAGCAAAAATCACAGCACCAGAGTCTAGATAATTTTCTGAACGCTTACACTTTCCAACTTCCCTCCGTACGCATCGCCCCCCGTCCCCCCCCCCCCCCCTTTTTTCTGTAAGCGCTGTAATTTTACGCTTCTGCTATGTTCAGGGCATTGAACTGCGATGATCACGGTGCAGCGGTACAACAAAATGCCTTCGGCTTTCCGAGGAAGTCTTCCGACCGTTGGACGGACAATTCAACCTCGAATTATCGGTGTGAAAGCGGAGGAAACGGTAGAGACAGCGGCCGCAATCGCGTCAGACGACCCATGAACGCATTCTTGGTGTGGTCTCGCGATCAAAGGCGCAAGATGGCTCTAGAGAATCCCCAAATGCAAAACTCAGAGATCAGCAAGCAGCTGGGGTACCAGTGGAAAATGCTTACAGAAGCCGAAAAATGGCCATTCTTCGAGGAGGCGCAGAGACTACAGGCCATGCACCGAGAGAAATACCCGGACTATAAATACCGACCTCGTCGGAAGGCCACGGCACAGAAAAGTCACAAATTGCTACCTGCAGCCTCCTCCTCCATGCTATGCAAGCAGGTGCACGTAGATGAGAGGTTCTACCCCTTCACCTACACGGACAGCTGTAGTAGGGCTGCACACACACGAATGGAGGACCAGTTAAGCTGCTCACAACCCATGAGCACAGCCAGGTCGCTGCTGCAACAGGAGTACCACAGCAGCTCCGCAAGCCTCCGTGACAGTCCGGAAACCTTGGCCGCACAGATGTCCGCTGACGCTTCTTTTTACTCTAAGTAACAGCTGGGACTTTCTGACGCTTATTTTCCTTAGTGATTTCTTTACTCTGGCTAACGAAACGTCCTATTCATACTGATTTTGCTATTATTTCATTCAAAATGAGCGTTGGAAACTTGCTTAACATATAAAGAATTACAAAGTATGCAATTGACTCAGGCTGGGTTTATGGCTGCTTAACTTGAGGAGAGAGCACGTTGATACTTTCTGAAACTCCTGGACTTCCAAGACCACTTGTTTTTCTTTTAACCAAACAACTGTTATATCTAATTATAGTTGCTCCATAGACTGAGCTTTAAATAAGTGAAATAATAGGTGCAAGAATATACTAATAATCTGATAATCGTCCTCATAGGTACCACAATTTTCACTCTTTATTTTTAAAATACTTCCTTTATTCATGAGAGACACCCAGAAAGAGGCAGAGACGTAGGCAGCGGGACAAGCAGGCTCCCTGCGGGGGGGAGACCGGTGGGGGACTCGCTGTCAGGAACCCCTCCCCCCCACCCCCCCATGGGGCCCTGAGCCAAAGTCCCACGCTCTACCCCTGAAGCCCCCGGGTGGACCTTCACTCTTTATTTTAAATAAATATTATTTATTATTATATTATTATATTATATTACATTATTTAAATAAATATTATAAATAAATATTTTTATCACA

**>Fragment UBE1Y6** (MG594255; Hellborg and Ellegren 2003, Fain and Straughan, unpublished)

**T**GTGGCAGCATCCAACCTCCGAGCAGAAAACTATAACATTCCCCCTGCAGATCGGCATAAGGTAATGATAGTCTAGAGCTAAGCTTTCACCCTCTCCCAAGGTTGAGTCTCCCAAATTTGTCCTCTTTTATAGATCCTGAACCTCTTTATAGACTGCATATGCTGGTCATCTTCTGTCCCTGCAGAGCAAGCTGATTGCAGGAAAGATCATCCCTGCCATTGCCACAACCACAGCAGCTATAGTTGGCCTCGTGTGTCTGGAGCTATACAAAGTGGTGCAGGGACACCAGCAGCTTGAGTCTTACAAGAACAGTTTCATCAACTTGGCC

**>Fragment UTY11** (Hellborg and Ellegren 2003, Fain and Straughan, unpublished)

**A**AAATTGTATTTGGTACACTGAACATTTAAGAGTCTTTAAATCTGCCAATAGTGGCATCATCTAAACATGTGCTAAATAGGCAGAGAGAGACAGAACTAAAAAAACTAAAAAGGGAAAAAAGTTTCTGCTAGTGTTTTAAAATATTAAAAATAGTAAAAGGTATTTCAAAGTTTATTTTTTCAATCTTACCTAACTTCAGAGCTCCAGCAAGGCCACGTATTACTGTAACTGGGTTTTTTGGATT

**>Fragment ZFY Intron** (AB622147; Tsubouchi et al. 2012)

GGCATCTCTTTACTATGCTTGGTTTTCACATGTGTTTTTAAGTTAGAAGAGTCTGCAGACCTATATTCGCAGTATTGGCACTGGTATGGCTTCTCGCCAGTGTGGATTCGCATGTGCTTTTTGAGCTCTGACGGGTGACGAAAACCTTTACCACACTCCACACAAATATGAGGAAAGTTCTTGCTGTGAACCGCCAAAAGGTGGCGATTCAATAAGCCTTGTTCAGCTGTCTCATATTCACAGAATTTACACTTGTGCATTTTGTTGGTTCCCTTTTCCTTATGCACCATTTTGTGTGTAAACAAAGCCCCAGCGTGAGAGAAATGCTTCCCACACTCCTCGCATTCGATAGACTTTTCTGCCTTGCTTGTTAGCTTGTGGCTCTCCAGGTGGTTGTGTAAACTTATCTTCTTATTTGTAGTGTAATCACAGTCAGTACAGCGGTACTTCTTCTTGGAAAGGTGTTCAGGATGGTTTTTCATGTGCCTTTTCAAAAAACCTCTCGACTTAAATTTTTTTCCACAAATCATGCAAGGATAGACAGTCAAGGGATGTCCATCAGGGCCAATAATTATTGCTAAGAAAGGGAAAGAAAAGAACATGAGTGATCAAACCAAGTTCTGCTTTGGTTCTTCAACCACTTAAAGTGTGTGCTCTGAAAATAGTGCTAAAGTACTCCTAAAAGCTAATATTCTTTCGTTTTTCAACAAAAGAGGATAGAAAAACCCAGTAATGAAGAAAATGTTGTTCAGAAACTTCATTCAATGTAACTACAAATCTCATTTCCAGTTTAAAAGACATTTAAAGCCATATATAGGGGTGTCTGGGTGGCTCAGTGGTTGATTTAGCAGTGCTAAATCTGCTTAGTTCTTAGTATCTGCCTTAGTTCAGGTCCTGATTCTGGGATTCTGGGATGGAGTCCTGCATGTGGCTCACTGCTGAGAGCCTGCTTCTCCCTTTGCCTACGTCTCTGCCTCTCTGTGTCTCTTATGAATAAGTAACAATAAAAAAGCCATATATACTTGGGAATCTATAGAAAATAATTCATAAAAACAATTCATAAAACCTTTCAGTTTCATTACTGCAAACCAAA

**>Fragment MS34A** (AF032441, Olivier et al. 1999, Sunqvist et al. 2001)

GATCCCTGGGAGGATTACTTTTCAACTCAGATTTAAGCATTCTCTTCCAGTTGGTCCAAGGCATGCTCAAAGAAATAGGTGCAACCTGTAGATGTGGCCTCTTCCCTTCTGGGAGATGGATAATATGCCACAGCCTGTTCGCTCTCGGCAGGCACTCCAATTTACCATGGGGATAATCTTGGATTGCCTCCCTAATTCCTTGGGATGGATAGTCGGTGCCAAGGAGAGTGTTACTAGCCATTCCTGGCCGAGTGGCCTCTCCTGTACCCCAGCTCTCCCATATCCATGTANNNNNNNNNNNNNNNNNNNNCACACATGCATCCATGTGTTCATATACACAATGATGGTTCATACAAAACTTTGGAATATCTACCCCACATCATCAGACACTATGGCAAAAGGGGACCTAGAGCATCCACTCGTCAACCTGATGACCTACCCTCAATGGAGCAAAGAATTCAATCAGAAGAACAAGAAAGTGTAGACAGCCACCCAACATCAAGTTCTTTGTCTTGTATTCACGAAAACTAACTTTACACTTAAGTAAAATGCTCAGCAATTTCTACATTTCTCGGACCTCTCAGCAGCCAGAGTGTCTACCAAAATATAAGTGCAAGAGGCTACATGGGATTACAGACAGCTAGTGTATGAAGGGATAATGTCAGGTGCTCATCTTGCCTTTCCTACTTGCTCTTTTGTGATACCTAGAACAATGATATGAAGGGTGGAGCTACAGCCACCTTTCTGGACCAAGAAGATTGGGAAGACTAGAACCATGGAACCATTGCCAACCCCCAGGTATTTCTGGACTTTTACTTCATGTTGAAAGTGAATACACTTCAGTCATTGTACACACTTACATCTGTGTGCTTCATGTTCTGTTATTAAGAGATGGATGTAGCTCATAACTATTACAGAAGATC

**>Fragment MS34B** (AF032441, Olivier et al. 1999, Sunqvist et al. 2001)

GATCCCTGGGAGGATTACTTTTCAACTCAGATTTAAGCATTCTCTTCCAGTTGGTCCAAGGCATGCTCAAAGAAACAGGTGCAACCTGTAGATGTGGCCTCTTCCCTTCTGGGGGATGGATAATATGCCACAGCCTGTTCGCTCTCGGCAGGCACTCCAATTTACCATGGGGATAATCTTGGATTGCCTCCCTAATTCCTTGGGATGGATAGTCGGTGCCAAGGAGAGTGTTACTAGCCATTCCTGGCCGAGTCCTCTCCTGTACCCCAGCTCTCCCATATCCATGTANNNNNNNNNNNNNNNNNNNNNNNNNNNNNNTGCATCCATGTGTTCATATTCACAATGATGGTTCATACAAAACTTTGGAATATCTACCCCACATCATCAGACACTATGGCAAAAGGGGACCTAGAGCATCCACTCGTCAACCTGATGACCTACCCTCAATGGAGCAAAGAATTCAATCAGAAGAACAAGAAAGTGTAGACAGCCACCCAACATCAAGTTCTTTGTCTTGTATTCACGAAAACTAACTTTACACTTAAGTAAAATGCTCAGCAATTTCTACATTTCTCGGACCTCTCAGCAGCCAGAGTGTCTACCAAAATATAAGTGCAAGAGGCTACATGGGATTACAGACAGCTAGTGTATGAAGGGATAATGTCAGGTGCTCATCTTGCCTTTCCTACTTGCTCTTTTGTGATACCTAGAACAATGATATGAAGGGTGGAGCTACAGCCACCTTTCTGGACCAAGAAGATTGGGAAGACTAGAACCATGGAACCATTGCCAACCCCCAGGTATTTCTGGACTTTTACTTCATGTTGAAAGTGAATACACTTCAGTCATTGTACACACTTACATCTGTGTGCTTCATGTTCTGTTATTAAGAGATGGATGTAGCTCATAACTATTACAGAAGATC

**>Fragment MS41A** (AF192268, Olivier et al. 1999, Sunqvist et al. 2001)

GTGGATCCTTTTTTACCTGGTGCACCTGATAATTGTCGAGGGTGTTAAAATAATCGAAGCCACAGACTGTCTCCTCCAATATCTCCTGGATGCGGCTCTACAAACACAGTGCCCTGTTTATAATCAAGAGACAATACGGTGCTCTCTGGACATCACCCAAGGGGACAGCCCCACTAGAAACTGTGTTCTCTAGTTAATGTCACAGGGGCAGCTGAAGAAACATCTGGCAAGGAGGAAGCCAGATGAAAGAGACTCCTGGGCTTATGATTAACTTATTGGAAGTTGAGCTTGGTCCCTAGCACTCACGTTCTCATCTTGCCTGTCATGACCTGGGGTTTGAACACAAAGATGTGTCAATCTTCCAACATCTGGCAGCTGGTTTCTGCCCTGGAAGTGTATATTCCTCTAATTTTCCCCTCTANNNNNNNNNNNNNNNNNNNNCACATACACACACAGATACACACACAGATGGGTAAAGGGTCTCACTCCACTCAGCTGTGATGGTAGTGTGGTCTGCTCACCAGTGAGCTGCCCCAAGTTGCCTTGCTGTACCTGTTGATGTCTCCTGACACATAACAAGAAGCTTCAGAGAAGAGGGTCGAGCAGGCGTACAATGACATGAAAATCCTCATTCTGGACTTTTCTGCACCCAGAGCACCTGAAAGAAGGAAACCAGCAACAGAGCAACCTGTTCTCTTAACCATGTTGAGTTAAATGAGCTGGAAAGCAGGAGGTTCTAGAATGCAGGGTCCTGGTTACCTGAGTTCTAAAGCAGTTGTGGATGTCACCAAGGAAGTGACGTCACTTTTTCAAGATTCCAGCATCTGGGGCTCTTCTTTCAGTCAGACCTATCCAGAACCCCTTCTTGCAGATGACTGCTTACCCCTGTCGTCTCCTTAACTGCCTCATCAGCCAACAAGGCCATTGCCATAACTCTCTGTTAATTCCAGTAAGGTCCTAACTTTTAGGAGACAGAGCTGAATCCAGACCTTTAGTTTTCTGCTTGGCTTCTCAGTTCTGTGTTGTATCTAACTCACGATGTCTGTCATATAGGTCCCTGTTTCCCAATTAGCACAGTGGTAGAATCAGA

**>Fragment MS41B** (AF192268, Olivier et al. 1999, Sunqvist et al. 2001)

GTGGATCCTTTTTTACCTGGTGCACCTGATAATTGTCGAGGGTGTTAAAATAATCGAAGCCACAGACTGTCTCCTCCAATATCTCCTGGATGCGGCTCTACAAACACAGTGCCCTGTTTATAATCAAGAGACAATACGGTGCTCTCTGGACATCACCCAAGGGGACAGCCCCACTAGAAACTGTGTTCTCTAGTTAATGTCACAGGGGCAGCTGAAGAAACATCTGGCAAGGAGGAAGCCAGATGAAAGAGACTCCTGGGCTTATGATTAACTTATTGGAAGTTGAGCTTGGTCCCTAGCACTCACGTTCTCATCTTGCCTGTCATGACCTGGGGTTTGAACACAAAGATGTGTCAATCTTCCAACATCTGGCAGCTGGTTTCTGCCCTGGAAGTGTATATTCCTCTAATTTTCCCCTCTCNNNNNNNNNNNNNNNNNNNNNNNNNNNNNNNNNNNNNNNNNNNNNNNNNNGATACACACACAATGATGGGTAAAGGGTCTCACTCCACTCAGCTGTGATGGTAGTGTGGTCTGCTCACCAGTGAGCTGCCCCAAGTTGCCTTGCTGTACCTGTTGATGTCTCCTGACACATAACAAGAAGCTTCAGAGAAGAGGGTCGAGCAGGCGTACAATGACATGAAAATCCTCATTCTGGACTTTTCTGCACCCAGAGCACCTGAAAGAAGGAAACCAGCAACAGAGCAACCTGTTCTCTTAACCATGTTGAGTTAAATGAGCTGGAAAGCAGGAGGTTCTAGAATGCAGGGTCCTGGTTACCTGAGTTCTAAAGCAGTTGTGGATGTCACCAAGGAAGTGACGTCACTTTTTCAAGATTCCAGCATCTGGGGCTCTTCTTTCAGTCAGACCTATCCAGAACCCCTTCTTGCAGATGACTGCTTACCCCTGTCGTCTCCTTAACTGCCTCATCAGCCAACAAGGCCATTGCCATAACTCTCTGTTAATTCCAGTAAGGTCCTAACTTTTAGGAGACAGAGCTGAATCCAGACCTTTAGTTTTCTGCTTGGCTTCTCAGTTCTGTGTTGTATCTAACTCACGATGTCTGTCATATAGGTCCCTGTTTCCCAATTAGCACAGTGGTAGAATCAGA

**>Fragment 35.4** (AY466399, Bannasch et al. 2005)

TCTACCTTTCCTCCATCCGCAGAACAGGTATTACTTAGAACTCCAGGCAGTAATACAAATGAAGCACCTGCCGTGTGTGTGTGTGTGTGTGTGTGTCTGTCTGTCTGCTCAATTTCTCTATCACCTAAAATAGCGGCCCTTCAAATATATCCAAAGACTAGGTCTCCAGGTGTGGAAGGACAGAGCTACACAGAACCTGGCAATGGATTAAACTGGGGAACTGAGGAAGAAGGAAGAATCAAGGATGTATCCAAATGCCTGACAAAGGTAATCAAGCAGAAGAATATCAAGATGAGTCTGT

**>Fragment 79.2** (AY466397, Bannasch et al. 2005)

TTTTTGGGTTTTTATAAAATACATCAACAGCCTTATCTACCTTCCCTGCCAAGATATTGGACAGATGTATGTCAAATGAGGATAACTCTCAGGAGCATGGTTTATTCTAGTCTTTACTTTTCCACTCAGACTTAAGCATTCTATTCCATTTGGTCCAGGGCAGGGGAAAGGAAACAGGTGCAACCTGCAGATATGGCCTCTTCTCTCCAGGGAACTGGAGAATATGCAACAGCCTGTTGGCTCTTGGGGAGCCCTCCGATTCACCATGAATATAATCTGGGAATGCCTCCCCAATTCCTTGGGGTGGAGTGCCAGTCCCTATGGAAAGTGGTGTTATCCAGTCTTGGTCATCAGGTGACGCCCCTGCACTCAGCTCTCCCTTGTCCATGNNNNNNNNNNNNNNNNNNNNNNNNNNNNNNCACATCCATGTGTGCACACAATGATGGCTCACACAAAACTTAGAAATATATACCCCACATTATATGACACTCTGAGACCTGCCCTCAACT

**>Fragment 79.3** (Y466398, Bannasch et al. 2005**)**

CTCCCTGGACATCACCCCAAGAGGATGCCACATTAGAAACCATGTTCTCCAGTTCAGCCCAGAGGGGCACCTTAAGAAATATTTGACAAGGAGGAAGCCATATGAAAGAGACTCTTGGGTTCATGACTAACATAGTCAGGGTTGAGCTTGTTCCCTAGCACACGCCTTCTCATCTTGCCTGCTATGACCTGGAGTCTGAAAACAAAAGATGTCCAATACCTGGCAGTTAGTTTCTGCCCAGGAAGGACATTTTCCTCTCCTCTGTACCCTGTAGACAAGCAAAGACACAAANNNNNNNNNNNNNNNNNNNNNNNNNNNNNNCACAGTGAGGGGTAAAAAGTTTCAAACCGCTCAGCTGAGATCCAATGACCAACCAAAGTTTTTTTTTCCAACCAAAGTTTTACTTGATGTTTTACAAAATTTTTAAGGATATTTATTAATTTCTTGAAGATAGAGACTAAGCAGGGTGAGCAGAAGAGGGAGCGAGAGAATCAGGGAGCCCAGTATAGGGTTCTAACCCTGGATTCTGGATCAGTGCTGTAATGAGGTGAGAATGGAAAGAGGGATCTT

**>Fragment 650** (AF005413, Olivier and Lust 1998)

GTCCTGGGTTGAAGCCCTACATTGGGATCTCTGCTCAGTGGGGAGCTGCTTCTCCTGCTCCTTTTGCTGCTCACTCTACTGATTCTCTCATTCTCTCTCCAAGAAATTAATAAATACCCTTAACAAGTTTTGGTAAAATATCAAGGAAAATTCTGATTGCTGTTTGGATCCAGAAGCAAAACCTTGATACCTCTACAGAGCACTTAAGGAGACAAAAGGGATTTTGGCTGAGGAGTGACTCTCAGAGTCCAGAATCTCATAGATCTCTTGATTTCCATTGAGAGGGCTCATTCGAGGACTTCCTGTGATGAGGCAACTGAGTCAAATGGAGCACCAGATGGAGTCCCTAACCCGAAACTGGTCCCTAACCCTAACCTGAACACAAACCCCAACTTGATCCCTAATACCTAATCTAACATGAACCTTGACCCTAACCCTAATATGAGCCATAATACAAACCAGACCCAAACTCAAAACCCAACCCTAATCCAAACTCTAACCTGAACTTTAGTCAAACCCTAACCTAAAACCTAATCTGACCCAAACCTGAAAAAGAAACCATAAACCTAAACCTAACCCTAAACCCTAATCCTTAACCCTAACTAGAACAAGTGCCCTATTCTGAACCCCAAGTCTAACACTAACCCGAACCCAGGAC

**>Fragment 990** (AF005414, Olivier and Lust 1998)

GTCCTGGGTTCTCCATTGTGTTCCACTGATCTGTGTGGCAACTTTTGTGCCAGTACCATATCCTTTTGATGATGACTACAGCTTTATAATATAGTTTGAAAACTGGAATTGTGATGCCTCCAGGATTGGTTTCCTTTTTTAACACTCCTCCGGCTATGTCAGGGTGTTTTCTGGTTCCATGCAAATTTTAGGGTTGTTTGTTCCAGGTCTGTGAAAAATGTCCATGGGATTGCATTGAATGTGTAGATTGCTTTCGGTAGTACAGACATTTTTACAATATTTGTCCTTCAGATCCATGAACATGGAATCTTTTTCCATTTCCTTGTGTCATCTTCAATTTCTTTCATTAGTGCTTTATAGTTTTCAGAGTACAGATCGTCTACCTCTTTGGTTAGGTTTGTCCCTAGGTGTCTTATGGGTTTTGGTGCAATTGTAAGTGGAACTGATTTCTTGGTTTCTCTCTCTGCTGCCTCATTATTGGGGTATAGAAATGCAACAGACTCTATACCTTGATTTTGTTATCCCGCAACTTTACTGAACTCAGGTATTAATTGTGGGAATTGTTTGGAGGAGTCTTTTAGGTTTTCTACATAGAGTATCATGCCACCTGTGAATAGTGGAGGTTTGACTTCTTCCTTGATGATTTGGAAGTCTTTTATTTCTTTGTGTTTTGTTGTTGTTGTGACTATGACTTCTGGTCCTACATTAAATAACTGTGGTGAGAGTGGACATTCCTGTCTTATTCCTGACCATAGAGGAAAAGCTCTTGGTTTCTCCCCACCGAGGATGACATTAGCTGTGAGTCTTTCCTAAACGGCCTTTATGATGATGCGGTATGTTCCCTCTATCCCCACTTTCTAGAGGGTTTTTTTTATCAAGAATAGATGCATCTACTGAGGGAACCATATAGTCCTTATCCTATCTTTTATTAAAGTGGCGTATCATGTTGATCGATTTTGAAATATTGAACCAACCCTGCAACCCAGGAC

**>Fragment AMELY Exon** (KC763835, Yan et al. 2013)

GGCACCCTGGTTATATCAACTTCAGCTATGAGGTAATTTTTCTCTTTACAAATTTTTTTTCTTTTTTTTTTTTCTTTACAAATTTTGACCATTGTTTGAGTTAACAATGCCCTGGGCTCTGCAAGAATAGTGTATTGATTCTTTAAGACATTTCTTAGTCCCACCTTTTCAGTTTATATTACCAGATTTCTCCTTTGATGAGTTGCCTCAAGCCTGCATTGCCTCTGCACACTCTTACTTGGCCTCTCTGACTCTGTGTCTCCTTCTTAAATGGCTATGAAGTTATTTATCATAAACTACTGCTCAGGGTGTGGCTGCATAGTAGGACAGAAAGTAAACTCTGGCTGAAGAGCTTTGTTCTATTCTAGTCCTATAAAAGATGGGGGAGTCAAGTCAAGTCAAGTTATTTAGGTTCCTTTCCAGCTAGAAACTCATGATTCTAAGATTTTCACAGTCTGTGTCTCCCTTCTTTGTCTTTACTGAAAGATCAATGATCAATAAGTCTATGAGACATTCTCACAGAAATGGAAAGCATGAGGCTCTCCTTAGCAATATACTAACCACAGGAAGTTGGGCAAATCATTTTTGTCTCAGGAATTTTGGTTTCCTCATCTGAAGAAAGGAAATTGTTTTAAATGCCCACACTTCAAATAGTTTGAGGAGCAAAATAGTTAAGTAATATAAAAAGTTCTTCATCAGATATAACTTTAGCAAAGTTATAATTGTTTCTTGTACTGATATAAAATGCTATACTACTACTTGAAGGCATCTTTCATCTCGTTAACCTTTTCACTATGACATAAAGACAAAAAGGTGTATAATTAATTTTATATCACATTAATTTTTTGTTTTGTTTTGGTCTTGCAAAGAAGAGCAGGAGCTATGGAATTAAACAAAAACACCCCAGTAAGACTATGAACTTCCTTAATTCCTCATGACTTTGTCTGTAAAAATGACAGTGTTGATATTTGTCTCAGCATATATGAGAGAGCATGAAATGTGTATGTGATTGACATAATACACTGCCTAACACATAAGAAGTACCTGGGAAATGTTTACTTTATTCTTTCTTCTGTAGAACTCAATTTTCAGACTATCAATATTGATGAGACTTTAATAGTTTGTATTTTATGCAGCTAAATTAAGGCAAATGAATTCTAACATCTCTTTCTCTTAAGGTGCTTACACCTCTGAAATGGTACCAGAATATAAGGCATCCGGTATGCAGACTTTTTGTCCTTTATTCCCTTAGAATATAAAATGTATTTCAATTTTCTTTTAAGTGAAATAATGTACCTATGCCACATACAGATCCTAATGGGAAATCTAGTTTGTAAAATGTCATATCTATGTGCACAGTTAGAAATTTCTCTACAAGGGAAAGTGGATAAATATTCATACTATCATAATGACAAAGAAAACATGACTATATCTCCTGTTGGAAGTCAGTTTGACTGAGTCACTGTAACTGAGTCAGTTGTGTGAGCTAATGATGAACCTGATTCTTTGTTTCCCACCAGTACCCTTCCTATGGTTACGAACCCATGGGTGGTTGGCTGCACCACCAAATCATTCCCATGCTGTCCCAGCAAAATCCCTCGAATCAAGCCCTGCAGCCTCATCATCACATCTCCATGGTGCCAGCTCAGCAGCCTGTGGTCCCCCAGCAACCAATGATGCCAGTTCCTGGCCAACACTCTATGACTCCAACTCAACATCACCAGCCAAACCTCCCTCTGCCTGCACAGCAGCCCTTCCAGCCACAGCCCGTCCAGCCACAGCCTCACCAGCCCATTCAGCCACAGCCACCTATGCACCCCATCCAGCCCCTGCTACCACAGCCACCTCTACCTCCGATGTTCCCCATACAGCCCCTTCCCCCCATGCTTCCTGACCTGCCACTGGAAGCTTGGCCAGCAACAGACAAGACCAAGCAGGAAGAAGTGG

**>FRAGMENT ZFY Exon** (JX475923, Gilbert and Silversides, unpublished)
ATGGTGGCTCCTCCGGAATGACCATCGACACGGAGTCCGAAATTGATCCTTGTAAGGTGGATGGCACTTGCCCTGAAGTCATCAAGGTATACATTTTTAAAGCTGACCCTGGAGAGGATGACTTAGGTAAGAAGAAAGCTTCAGCATATTATACATCCTGATCAGATACACTTCAATTTGATTTCTTTTGGGTGGTTTAGATTGAGGACGTTTGATACTTTTTCTCTTCTCCTCTTTTCTCTTTCTTGCTCTTTTTAGATCGTATTTATTTGTTTGACAGAGAAAACACAAGCAGGGAGAGCAGGAGAGGGAGAAACAGGCTCTCTGTTGAGCAGGAAGCCTGATGTGGGGCTCTGTGATTATGGGATCATGAACCAGGCCAAAAGCAGACTCACCGAAGTGACTGAGCTACCCAGGTGCCCCTGAAATCTTTTCTGAAGAGAACTTTTAAGCAAGAATTTTAGGGCAGCCCCGGTGGCTCAGCCCGGTTAGTGCCGCCTTCAGCCTGGGACATGATCCTGGAGACCCGGGATCGAGTCCCACATCGGGCTCCCTGCATGGAGCCTGCTTCTCCCTCTGCCTGTGTCTCTGCCTCCCTCTCTCTTTCTGTATCTCTCATTAACAAAAAATAATAATAATAATAATCAAGAATTTTATTGTGGCAGTAGAAAAATATATAGAAAGTGTAATAAGTTAAACAGGTGGAAATGAAGTTTTCACATTAAACTTGGAGCTGTCATCTTCCCTGATAGGTGGCACTGTGGACATTGTGGAGAGTGAGCCTGAGAATGACCATGGAGTTGAATTACTTGATCAGAATAGCAGTATTCGAGTTCCAAGGGAAAAGATGGTTTATATGACTGTTAACGATTCTCAGCAAGAAGATGAAGATTTAAGTAAGTAGGTGCCTTTGTTGTGGGAGAAAATTTTGTTTCTGTTTACAGTAATATATGAAAATAATGTTAATGAGTCTGTAACATTTGAAACTTGTTTGTCTGAATTCAAACAAGTATCTTTTTCTGTCTGACCTTTGTAAACCTGATTATAAACGTTTTACTAATTTTCTAATTAGATGTTGCTGAAATTGCTGATGAAGTTTATATGGAAGTCATAGTAGGAGAGGAGGATGCAGCAGTTGCAGCAGCAGCAGCAGCTGCTGTTCATGAACAGCAGATGGATGACAATGAAATAAAAACTTTCATGCCAATAGCATGGGCAGCAGCTTATGGTGAGTCACATACAGTAGCTCAAAGGATTGCATAGTTGTGGAATACGAATTCACCATTGAGAATAATTTCAGTAGTTTCAGATTTCAGAAATAGGAAGTATCGATATAGTGATTCAAGGACACAGAGAATGTATATTCTGCCTATTTTTGCCTTTCATTTTATTATTATTTTCAAGATTTTGTTTGTTTATGAGAGACACAGAAAGAAAGGCAGAGACATAGGCAGAGGGAGGAGCAGGCTCCCTGTGAGGAGTCTGATGTGGGACTTGATCCCAGGATCCCAGGATCACACCCTGAACCAAAGGCAGAAGTTCAACCAATGAGCCACCCAGGCATCTCTCCCTTTAATTTTAATAGGAATTTACTTCGCCTATGTTATCTAAGAGATAGACATTAAAGGTGGAATTTTTATCTTCAGTATCTGGGTCATGATTTTATAATTTTGAGTGTCATCAAGGTGAAAAATGCTGCTACATTTGGAAGCTAGGCTTTTCATGTTGTTCAGTACATAATATGCCCTTATAGCGCATTACGTGGATAGGGAGTTTGTCATTCATGAGTATCGTGGCTTTTCATTGTTATGGTTAATAAAGAATCCCTAATTCTTTATAAAGAATACTGTATAATATTGTTTTTTAATACACATTGTTAGGTAATAATTCTGATGGCATTGAAAACCGGAATGGCACTGCAAGTGCCCTCTTGCAC

**References**

Ding ZL, Oskarsson M, Ardalan A, Angleby H, Dahlgren LG, Tepeli C, Kirkness E, Savolainen P, Zhang YP. Origins of domestic dog in southern East Asia is supported by analysis of Y-chromosome DNA. Hered. 108:507-514. [see Supplement 1]

Bannasch DL, Bannasch MJ, Ryun JR, Famula TR, Pederson NC (2005) Y-chromosome haplotype analysis in purebred dogs. Mammalian Genome, 16, 273–280.

Gilbert K, Silversides DW (Unpublished) Canine ZFY DNA sequences for DNA sexing and forensic procedures.

Hellborg L, Ellegren H (2003) Y chromosome conserved anchored tagged sequences (YCATS) for the analysis of mammalian male-specific DNA. Mol Ecol. 12:283-291.

Meyers-Wallen VN, Schlafer D, Barr I, Lovell-Badge R, Keyzner A (1999) SRY-negative XX sex reversal in purebred dogs. Mol. Reprod. Dev., 53, 266-273.

Natanaelsson C, Oskarsson MC, Angleby H, Lundeberg J, Kirkness E, Savolainen P (2006) Dog Y chromosomal DNA sequence: identification, sequencing and SNP discovery. BMC genetics, 7, 45.

Olivier M, Lust G (1998) Two new nucleotide sequences specific for the canine Y chromosome. Animal Genetics, 29,146–149.

Olivier M, Breen M, Binns MM, Lust G (1999) Localization and characterization of nucleotide sequences from the canine Y chromosome. Chromosome Research, 7, 223–233.

Sundqvist AK, Ellegren H, Olivier M, Vila C (2001) Y chromosome haplotyping in Scandinavian wolves (Canis lupus) based on microsatellite markers. Molecular Ecology, 10, 1959–1966.

Tsubouchi A, Fukui D, Ueda M, Tada K, Toyoshima S, Takami K, Tsujimoto T, Uraguchi K, Raichev E, Kaneko Y, Tsunoda H, Masuda R (2012) Comparative molecular phylogeny and evolution of sex chromosome DNA sequences in the Family Canidae (Mammalia: Carnivora). Zool Sci 29:151–161.

Yan S, Bai C, Li Y, Hou J, Zhao Z, Han W (2013) Sex identification of dog by PCR based on the differences in the AMELX and AMELY genes. Animal Genetics, 44, 606.
